# PriorAug: Reliability-Aware Traffic Scenario Initialization Under Sensor-Perceived Structural and Interaction Priors

**DOI:** 10.3390/s26154869

**Published:** 2026-08-02

**Authors:** Kaixi Yang, Shiru Qu

**Affiliations:** School of Automation, Northwestern Polytechnical University, Xi’an 710072, China

**Keywords:** autonomous driving simulation, traffic scenario initialization, sensor-perceived priors, structural reliability, interaction reliability, prior augmentation, Waymo Open Motion Dataset, intelligent transportation systems

## Abstract

Traffic scenario initialization is a key component of autonomous driving simulation. The generated initial traffic states directly affect downstream motion prediction, planning evaluation, closed-loop simulation, and safety assessment. Existing traffic scenario generators usually rely on structural and interaction priors, but these priors are commonly treated as deterministic and fully reliable during both training and deployment. In practical autonomous driving systems, however, such priors are reconstructed from heterogeneous perception pipelines involving sensors, localization, tracking, online mapping, map alignment, and behavior estimation. Their reliability may therefore vary continuously because of sensing noise, localization drift, incomplete observations, occlusion, tracking instability, and imperfect interaction inference. To address this issue, this paper proposes PriorAug, a reliability-aware prior augmentation framework for robust traffic scenario initialization under sensor-perceived prior uncertainty. PriorAug formulates traffic scenario initialization as conditional generation over sensor-perceived priors and introduces a two-dimensional reliability space to characterize structural reliability and interaction reliability separately. Based on this space, reliability-conditioned prior operators are designed to construct diverse prior configurations during training, enabling the generator to learn from continuously varying perception conditions without modifying the underlying generation architecture or introducing additional learnable inference modules or iterative decoding stages. Experiments on the Waymo Open Motion Dataset show that PriorAug maintains competitive full-set distribution fidelity, improves robustness under shifted-prior conditions and traffic density variations, and improves the physical plausibility of generated initial states. Reliability landscape analysis further demonstrates that coverage-oriented prior augmentation yields smoother performance transitions across the reliability space. These results indicate that explicitly modeling sensor-perceived prior reliability provides an effective and practical route to robust traffic scenario initialization for autonomous driving simulation.

## 1. Introduction

High-fidelity traffic scenario initialization is a fundamental component of autonomous driving simulation. The initialized traffic state determines the spatial layout, motion attributes, and interaction context of surrounding traffic participants, and therefore directly affects downstream motion prediction, planning evaluation, closed-loop simulation, and safety assessment. Compared with manually designed scenarios, data-driven traffic scenario generation can learn from large-scale autonomous driving datasets and provide diverse traffic states with stronger statistical realism. Recent studies have developed various learning-based generation frameworks, including traffic scene generation, unified initial-state-and-trajectory modeling, controllable diffusion-based generation, and language-conditioned traffic generation [1,2,3,4,5]. These methods have improved the realism, diversity, and controllability of simulation scenarios.

Most existing traffic scenario initialization methods rely on structural and interaction priors to constrain generation. Structural priors, such as lane geometry, road topology, road boundaries, and driving directions, provide spatial constraints for vehicle placement and motion attributes. Interaction priors, such as following, yielding, merging, overtaking, and collision-avoidance relationships, describe behavioral dependencies among agents. By conditioning generation on these priors, traffic generators can produce more realistic initial traffic states than unconstrained generation. However, existing methods often treat these priors as deterministic, complete, and fully reliable during both training and deployment. It should be emphasized that this work does not question the necessity of structural and interaction information for autonomous driving. Instead, it focuses on the reliability variation of these priors after they are reconstructed by upstream perception and scene-construction modules. The deterministic-prior assumption is difficult to satisfy in practical autonomous driving systems. In deployment, the priors available to a traffic generation module are not perfectly annotated ground-truth inputs. Instead, they are reconstructed from upstream perception and scene-construction processes that may involve cameras, LiDARs, radars, localization, object detection, multi-object tracking, online mapping, map alignment, and behavior estimation. High-definition maps and online map representations may suffer from incompleteness, localization drift, road changes, registration errors, and update latency [6,7,8,9]. Vectorized scene representations and lane-graph encodings used by downstream models also depend on the quality of perceived road structures and agent states [10,11,12]. Therefore, the priors used by traffic scenario initialization are more appropriately regarded as *sensor-perceived priors* rather than deterministic ground-truth priors.

The uncertainty of sensor-perceived priors is not uniform. Structural information and interaction information originate from different components of the perception and inference process and may vary independently. Structural priors are mainly affected by sensing noise, localization drift, map alignment errors, incomplete observations, and road structure reconstruction quality. Interaction priors are inferred from dynamic object states and observed trajectories, making them sensitive to occlusion, tracking instability, traffic density, behavioral ambiguity, and imperfect interaction estimation. Thus, prior uncertainty should not be reduced to a single binary condition such as map available versus map unavailable. A more expressive formulation is needed to characterize the continuous and heterogeneous reliability changes of perceived structural and interaction information.

Such reliability variation creates an important source of robustness degradation. A traffic generator trained under one fixed prior configuration may perform well near its native operating point, but its generation quality can deteriorate when the reliability of structural or interaction priors shifts. This issue is particularly important for traffic scenario initialization, because initialization errors may propagate to downstream simulation and lead to unrealistic vehicle placement, distorted motion attributes, or physically invalid overlapping states. Although previous studies have improved generation fidelity under fixed or idealized input conditions, the effect of continuously changing sensor-perceived prior reliability remains underexplored.

This perspective is related to domain randomization and robust learning. Domain randomization improves deployment robustness by exposing a model to randomized environmental or simulation conditions during training. Robust reinforcement learning similarly studies how policies can remain stable under uncertain dynamics, perturbed observations, or distributional changes. PriorAug follows the same broad principle of training under diversified conditions, but adapts it to traffic scenario initialization rather than policy learning. The randomized factors in PriorAug are not simulator physics or control dynamics; they are the reliabilities of sensor-perceived structural and interaction priors. This distinction motivates the reliability space formulation used in this paper.

To address this problem, this paper proposes PriorAug, a reliability-aware prior augmentation framework for robust traffic scenario initialization. Instead of assuming deterministic structural and interaction priors, PriorAug explicitly models structural reliability and interaction reliability using two continuous variables, denoted by $r_{s}$ and $r_{i}$, respectively. These variables define a two-dimensional reliability space in which each point corresponds to a specific sensor-perceived prior condition. Based on this formulation, reliability-conditioned prior operators are designed to adjust structural and interaction information independently during training. By exposing the generator to diverse reliability configurations, PriorAug improves robustness under shifted-prior conditions without modifying the underlying generation architecture or introducing additional learnable inference modules or iterative decoding stages.

The main contributions of this paper are summarized as follows:

- We formulate traffic scenario initialization as a reliability-aware conditional generation problem over sensor-perceived priors, addressing the mismatch between deterministic-prior training assumptions and uncertain perception conditions in practical autonomous driving systems.
- We introduce a two-dimensional reliability space that separately characterizes structural reliability and interaction reliability, enabling different sources of prior uncertainty to be modeled explicitly.
- We propose PriorAug, a reliability-conditioned prior augmentation framework that constructs diverse prior configurations through structural and interaction prior operators, improving robustness under continuously varying perceived-prior conditions.
- We evaluate the proposed framework on the Waymo Open Motion Dataset under a unified protocol. Experimental results show that PriorAug maintains competitive full-set distribution fidelity, improves robustness under shifted-prior and traffic density variations, and improves the physical plausibility of generated initial states.

## 2. Related Work

### 2.1. Traffic Scenario Generation with Structured Conditions

Traffic scenario generation aims to construct realistic and diverse traffic states for autonomous driving simulation. Learning-based approaches have been widely studied because they can capture statistical regularities from large-scale driving datasets. SceneGen learns realistic traffic scene layouts from recorded driving data [1], while TrafficGen provides a representative framework for structured traffic scenario generation [2]. More recent methods extend traffic generation toward joint initialization and trajectory modeling, controllable generation, and semantic control. For example, UniGen jointly models initial agent states and future trajectories [3], SceneControl introduces diffusion-based controllable traffic scene generation [4], and language-conditioned generation explores textual or semantic guidance for traffic generation [5].

These studies show that structured conditioning information is essential for high-quality traffic scenario generation. However, the conditioning inputs used by existing generators are usually treated as reliable once they are provided to the model. In practical autonomous driving systems, such inputs are produced by upstream perception, localization, tracking, map-processing, and behavior estimation modules. Their reliability may vary with sensing quality, localization accuracy, observation completeness, and traffic complexity. Therefore, traffic scenario generation should not only consider what prior information is available, but also how reliable this information is under deployment conditions.

### 2.2. Sensor-Perceived Structural and Interaction Information

Structural information provides road-level constraints for autonomous driving perception, prediction, planning, and simulation. High-definition maps and online map representations provide lane geometry, road topology, road boundaries, and driving directions, but they may be affected by incompleteness, update latency, localization drift, registration errors, and maintenance cost [6,7,8]. Road change detection and HD map registration studies further indicate that aligning perceived road structures with prior maps remains challenging under real-world conditions [9]. In learning-based autonomous driving models, vectorized scene representations and lane-graph encodings are also widely used to represent structured road information [10,11,12]. These representations demonstrate the importance of structural priors, but their quality still depends on perception and scene-construction reliability.

Interaction information provides another important source of traffic context. Multi-agent behavior is shaped not only by road structure but also by dynamic relationships among surrounding participants. Social GAN models socially acceptable trajectories through generative adversarial learning [13], Trajectron++ represents heterogeneous multi-agent interactions in a dynamically feasible prediction framework [14], and AgentFormer captures socio-temporal dependencies using agent-aware transformers [15]. Although these studies demonstrate the value of interaction modeling, interaction cues in practical systems are inferred from detected objects, tracked trajectories, and behavior estimation modules rather than directly observed as ground-truth. Therefore, interaction information may have reduced reliability under occlusion, dense traffic, tracking instability, and behavioral ambiguity.

These observations motivate separating structural reliability from interaction reliability. Structural priors and interaction priors originate from different upstream processes and may fail in different ways. A traffic generator that assumes both types of information to be fully reliable may become sensitive to reliability shifts during deployment. This paper explicitly models structural and interaction reliability as two independent dimensions of sensor-perceived prior uncertainty.

### 2.3. Robust Generation Under Prior Reliability Shift

Existing traffic generation methods commonly improve robustness through larger datasets, stronger conditional encoders, or richer contextual representations. However, their structural and interaction conditions are generally treated as reliable once provided to the generator, and few studies explicitly characterize how generation quality changes when the reliability of these conditions shifts. This limitation is important for traffic scenario initialization, because errors in perceived structure or interaction may directly affect generated positions, headings, speeds, sizes, and geometric feasibility. PriorAug addresses this gap by independently controlling structural and interaction reliability and training the generator over a two-dimensional reliability space rather than at one deterministic operating point.

### 2.4. Domain Randomization and Robust Learning

The training strategy of PriorAug is related to the broader methodological paradigms of domain randomization and robust learning. Domain randomization has been widely used to improve sim-to-real transfer by randomizing visual, environmental, or physical parameters during training, so that the learned model is less likely to over-specialize to a single source-domain condition [16,17,18]. Related ideas have also been explored in robust reinforcement learning, where policies are trained or optimized to remain effective under uncertain dynamics, model perturbations, observation noise, adversarial disturbances, or distribution shifts [19,20]. The common principle behind these methods is to improve deployment robustness by exposing the model to a family of possible operating conditions during training rather than to one fixed condition.

PriorAug follows this general principle, but adapts it to the specific problem of traffic scenario initialization under sensor-perceived prior uncertainty. Unlike conventional domain randomization methods that randomize simulator textures, physical parameters, or transition dynamics, PriorAug randomizes the reliability of the structural and interaction priors used by a traffic generator. Unlike robust reinforcement learning, PriorAug does not learn a control policy or optimize closed-loop behavior under uncertain dynamics. Instead, it trains a conditional traffic initialization generator to remain robust when the reliability of its sensor-perceived prior inputs changes.

The specific novelty of PriorAug lies in its reliability space formulation. Rather than treating prior perturbation as an unstructured input dropout or a single degraded condition, PriorAug defines a two-dimensional reliability space that separately characterizes structural reliability and interaction reliability. This formulation allows road structure information and agent-interaction information to be controlled independently during training. Therefore, PriorAug can be viewed as a reliability-aware adaptation of the domain-randomization principle to traffic scenario initialization, where the randomized factors are not generic simulator conditions but the reliability of sensor-perceived priors.

## 3. Proposed Method

This section presents the proposed PriorAug framework. We first define the sensor-perceived scene graph used by the traffic generator. We then introduce a two-dimensional reliability space to characterize structural and interaction reliability. Based on this space, reliability-conditioned prior operators are designed to construct diverse perceived-prior configurations during training. Finally, we describe the reliability-aware generator and the PriorAug training strategy.

**Figure 1:**
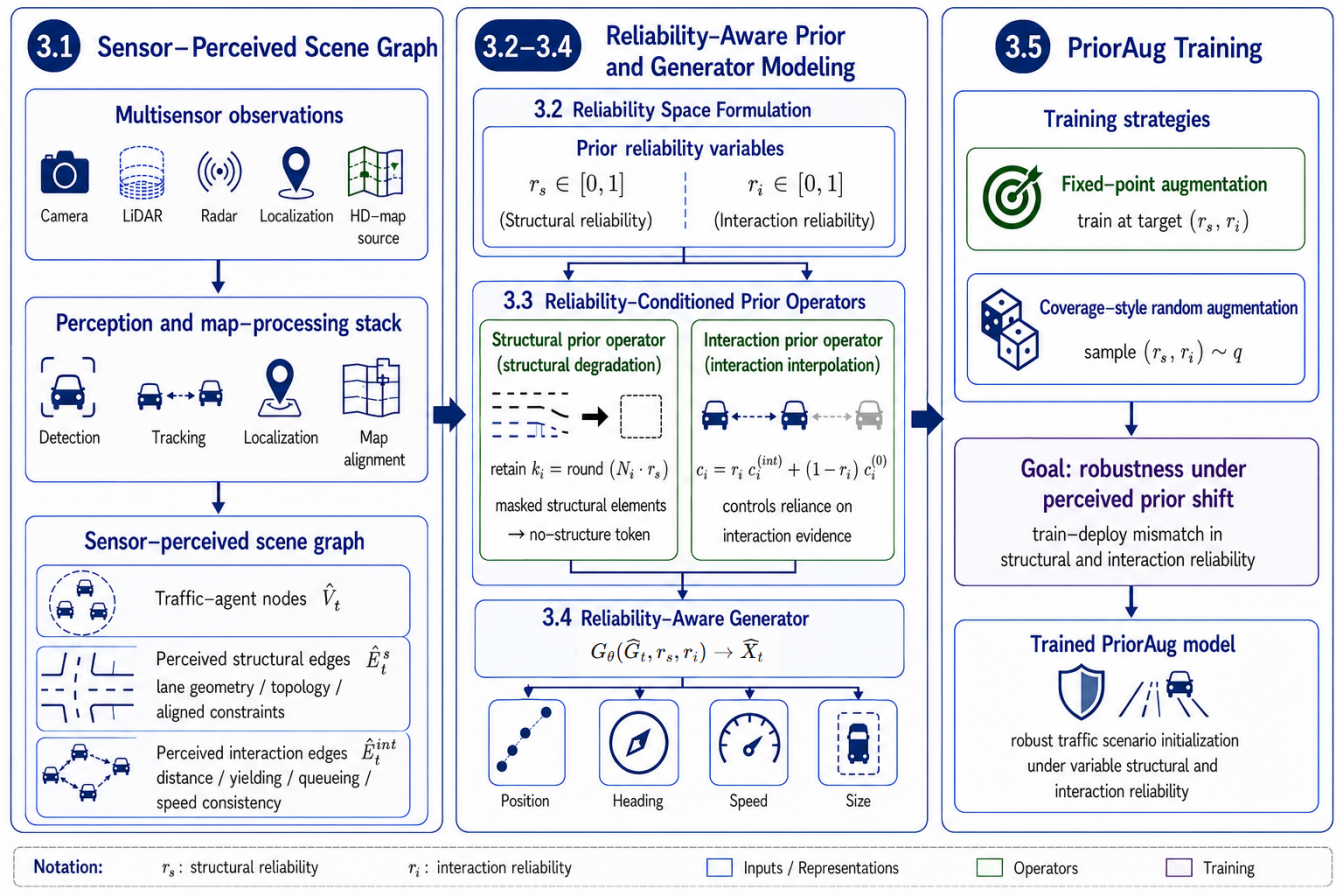
Overall framework of the proposed PriorAug. The framework consists of sensor-perceived scene-graph construction (Section 3.1), reliability space formulation and reliability-conditioned structural and interaction prior modeling (Section 3.2, Section 3.3 and Section 3.4), and fixed-point or coverage-oriented reliability augmentation during training (Section 3.5).

Figure 1 summarizes the overall reliability-aware training framework. The upstream scene-construction process provides a sensor-perceived scene graph. The reliability-conditioned prior operators then adjust structural and interaction information according to sampled reliability variables. The resulting scene graph is fed into the traffic generator, enabling robust traffic scenario initialization under continuously varying sensor-perceived prior conditions.

### 3.1. Sensor-Perceived Scene Graph

Traffic scenario initialization relies on structured priors that describe road geometry and traffic participant relationships. In practical autonomous driving systems, such priors are not directly obtained as perfect ground-truth inputs. Instead, they are produced by upstream perception and scene-construction processes involving sensor observation, localization, object detection, tracking, map alignment, online map construction, and behavior estimation. This paper does not explicitly model the full perception network itself. Rather, we abstract its downstream output as a sensor-perceived prior and study how reliability variations in this prior affect traffic scenario initialization.

Let

(1) $$P_{t}=\Phi \left(O_{t}\right),\;O_{t}=\left\{I_{t},L_{t},R_{t},l_{t}\right\},$$

where $I_{t}$, $L_{t}$, $R_{t}$, and $l_{t}$ denote camera images, LiDAR measurements, radar measurements, and localization information, respectively. Here, Φ(·) abstracts the upstream perception and scene-construction process; the present work studies its structured output rather than implementing the complete perception pipeline.

The abstract sensor-perceived prior $P_{t}$ is represented in the subsequent formulation as the scene graph $G_{t}$.

We represent the sensor-perceived prior as a scene graph:

(2) $$G_{t}=\left(V_{t},E_{t}^{s},E_{t}^{i}\right),$$

where $V_{t}$ denotes traffic-agent nodes, $E_{t}^{s}$ denotes structural edges, and $E_{t}^{i}$ denotes interaction edges. Each node $v_{k}\in V_{t}$ contains the state attributes of one traffic participant, including position, heading, speed, size, and semantic category. Structural edges encode perceived road-level constraints such as lane geometry, road topology, driving direction, and local road connectivity. Interaction edges encode inferred behavioral relationships among agents, such as following, yielding, merging, overtaking, and collision avoidance.

Because both $E_{t}^{s}$ and $E_{t}^{i}$ are derived from perceived scene information, they may be incomplete, noisy, or partially unreliable under practical sensing conditions. This motivates modeling their reliability explicitly rather than treating the scene graph as a deterministic input.

### 3.2. Reliability Space Formulation

The uncertainty of sensor-perceived priors does not affect all information equally. Structural information and interaction information originate from different upstream processes and therefore exhibit different reliability characteristics. To model this difference, we introduce two continuous reliability variables.

**Structural reliability.** Structural reliability measures the completeness and correctness of perceived road structure information. It is denoted by

(3) $$r_{s}\in \left[0,1\right],$$

where $r_{s}=1$ indicates fully reliable structural information, and $r_{s}=0$ indicates that structural information is unavailable.

**Interaction reliability.** Interaction reliability measures the confidence of inferred behavioral relationships among traffic participants. It is denoted by

(4) $$r_{i}\in \left[0,1\right],$$

where larger values indicate more reliable interaction information.

Together, the two variables define a two-dimensional reliability space:

(5) R=[0,1]×[0,1].

Each point $(r_{s},r_{i})\in R$ corresponds to one sensor-perceived prior condition. Methods relying mainly on structural priors can be interpreted as operating near high structural reliability and low interaction reliability, while methods using both structural and interaction priors operate near the upper-right region of the reliability space. In practical deployment, however, sensing quality, localization accuracy, tracking stability, and behavior estimation confidence may change over time, causing the operating condition to move continuously within this space.

**Figure 2:**
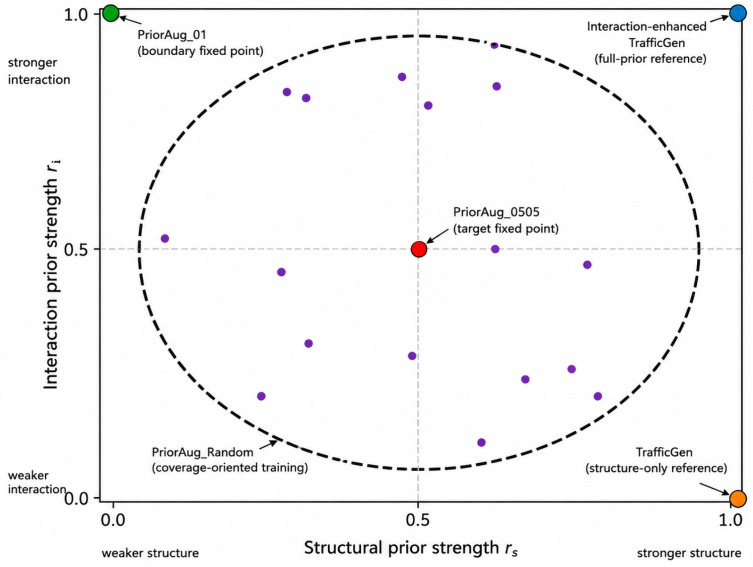
Two-dimensional reliability space of sensor-perceived priors. The horizontal and vertical axes denote structural reliability $r_{s}$ and interaction reliability $r_{i}$, respectively. The expressions “structural prior strength” and “interaction prior strength” used in the schematic are visual descriptions of structural and interaction reliability, respectively. The corner and central points illustrate representative deterministic operating conditions. The dashed ellipse schematically indicates the coverage region, and the purple circles denote sampled interior reliability conditions used by PriorAug_Rand. The label PriorAug_Random in the schematic corresponds to PriorAug_Rand in the text and tables. TrafficGen is shown conceptually near (1,0) as a structure-only reference; in implementation tables, its interaction reliability is marked as N/A because it contains no explicit interaction-control branch.

Figure 2 illustrates the proposed reliability space. Instead of optimizing traffic generation for a single deterministic operating point, the objective of this work is to learn a generator that remains robust across different structural and interaction reliability conditions.

### 3.3. Reliability-Conditioned Prior Operators

To instantiate different reliability conditions during training, we introduce two reliability-conditioned prior operators. The structural operator adjusts the availability of perceived structural information, while the interaction operator adjusts the strength of inferred interaction information.

#### 3.3.1. Structural Prior Operator

Given structural edges $E_{t}^{s}$ and structural reliability $r_{s}$, we generate a binary mask for structural elements:

(6) $$M_{t}^{s}\left(e\right)∼\mathrm{Bernoulli}\left(r_{s}\right),\;e\in E_{t}^{s}.$$

The reliability-adjusted structural edges are then obtained as

(7) $${\tilde{E}}_{t}^{s}=M_{t}^{s}⊙E_{t}^{s},$$

where ⊙ denotes element-wise masking. When $r_{s}=1$, all structural information is preserved. When $r_{s}=0$, structural information is removed. Intermediate values correspond to partially perceived structural conditions.

Here, $M_{t}^{s}\left(e\right)$ is a Bernoulli variable indicating whether structural element *e* is retained. Each element is preserved with probability $r_{s}$ and masked with probability $1-r_{s}$; therefore, $r_{s}$ controls the expected retained proportion of structural information. The mask is applied before the structural representation enters the generator and does not modify the backbone architecture or decoding procedure.

#### 3.3.2. Interaction Prior Operator

Interaction information is inferred from dynamic traffic context and may degrade gradually rather than disappear abruptly. In this work, interaction reliability is controlled at the context-representation level after the interaction information has been encoded and aggregated by the interaction module. This design avoids directly deleting raw agent features and allows the generator to smoothly adjust the additional contribution introduced by explicit agent–agent interaction aggregation.

Let $c_{i}^{\left(0\right)}$ denote the sample-specific base context obtained by bypassing the explicit agent–agent interaction aggregation module, and let $c_{i}^{\left(\mathrm{int}\right)}$ denote the interaction-enhanced context produced when the interaction module is fully enabled. The interaction-neutral endpoint used in this work is therefore $c_{i}^{\left(0\right)}$. It is not a zero vector, a dataset-wide average representation, or a learnable neutral embedding.

The interaction-induced context increment is defined as

(8) $$∆c_{i}^{\left(\mathrm{int}\right)}=c_{i}^{\left(\mathrm{int}\right)}-c_{i}^{\left(0\right)}.$$

Given the interaction reliability $r_{i}\in \left[0,1\right]$, the reliability-adjusted interaction context is constructed as

(9) $${\tilde{c}}_{i}\left(r_{i}\right)=c_{i}^{\left(0\right)}+r_{i}∆c_{i}^{\left(\mathrm{int}\right)}.$$

Equivalently, this can be written as

(10) $${\tilde{c}}_{i}\left(r_{i}\right)=r_{i}c_{i}^{\left(\mathrm{int}\right)}+\left(1-r_{i}\right)c_{i}^{\left(0\right)}.$$

When $r_{i}=0$, the explicit interaction aggregation contribution is removed while the base context is retained. When $r_{i}=1$, the complete interaction-enhanced context is used. Intermediate values provide a smooth transition over the interaction reliability dimension by scaling the interaction-induced context increment.

The base context $c_{i}^{\left(0\right)}$ is used as the interaction-neutral endpoint because it preserves sample-specific non-interaction information and the decoder input dimensionality. In contrast, a zero vector would remove both interaction and non-interaction context, a dataset mean would lose sample specificity, and a learnable embedding could implicitly encode interaction patterns. Thus, $r_{i}$ controls only the additional context introduced by explicit interaction aggregation.

#### 3.3.3. Unified Reliability-Conditioned Prior

After applying the structural and interaction prior operators, the generator receives a reliability-conditioned prior representation. The structural operator produces the reliability-adjusted structural representation ${\tilde{E}}_{t}^{s}$, while the interaction operator produces the reliability-adjusted interaction context ${\left\{{\tilde{c}}_{i}\left(r_{i}\right)\right\}}_{i=1}^{N}$. We denote the resulting reliability-conditioned prior representation as

(11) $${\tilde{G}}_{t}^{\left(r_{s},r_{i}\right)}=\left(V_{t},{\tilde{E}}_{t}^{s},{\left\{{\tilde{c}}_{i}\left(r_{i}\right)\right\}}_{i=1}^{N}\right).$$

Here, $V_{t}$ denotes the traffic-agent nodes, ${\tilde{E}}_{t}^{s}$ denotes the structural information adjusted by the structural reliability $r_{s}$, and ${\tilde{c}}_{i}\left(r_{i}\right)$ denotes the interaction context adjusted by the interaction reliability $r_{i}$. This notation is used to represent the complete reliability-conditioned input to the traffic generator. The interaction component should be understood as the context representation obtained after interaction encoding and aggregation, rather than as a raw edge-level interpolation. *N* denotes the number of traffic-agent nodes in the scene.

Each pair $\left(r_{s},r_{i}\right)$ therefore defines one realization of the sensor-perceived prior condition. By varying $r_{s}$ and $r_{i}$, the training process exposes the generator to a broad range of structural and interaction reliability configurations. This enables the model to learn traffic scenario initialization under continuously varying perceived-prior conditions rather than under a single deterministic operating point.

### 3.4. Reliability-Aware Generator

Given the reliability-conditioned prior representation ${\tilde{G}}_{t}^{\left(r_{s},r_{i}\right)}$, traffic scenario initialization aims to generate the initial states of traffic participants:

(12) $$X_{t}=\left\{x_{1},x_{2},\ldots ,x_{N}\right\},$$

where each $x_{k}$ contains the generated position, heading, speed, size, and category of one traffic participant.

Instead of learning a deterministic mapping from a fixed scene graph

(13) $${\hat{X}}_{t}=F_{\theta }\left(G_{t}\right),$$

Here, θ denotes the parameters of the traffic generator.

PriorAug learns a reliability-aware conditional generator:

(14) $${\hat{X}}_{t}=F_{\theta }\left({\tilde{G}}_{t}^{\left(r_{s},r_{i}\right)},r_{s},r_{i}\right).$$

Equivalently, the model learns the conditional distribution

(15) $$p_{\theta }\left(X_{t}|{\tilde{G}}_{t}^{\left(r_{s},r_{i}\right)},r_{s},r_{i}\right),$$

which allows generation quality to adapt to different perceived-prior reliability conditions.

### 3.5. PriorAug Training

PriorAug modifies the perceived prior during training rather than changing the backbone generator architecture. For each training iteration, a reliability pair is sampled from a predefined reliability sampling distribution:

(16) $$(r_{s},r_{i})∼q,$$

where *q* denotes the training distribution over the two-dimensional reliability space. Two strategies are considered.

**Fixed-point PriorAug.** The reliability pair is fixed at a predefined operating point, such as (0,1) or (0.5,0.5). This strategy can be regarded as sampling from a degenerate distribution concentrated at the target reliability point:

(17) $$q\left(r_{s},r_{i}\right)=\delta \left(r_{s}-r_{s}^{*}\right)\delta \left(r_{i}-r_{i}^{*}\right).$$

Here, δ(·) denotes the Dirac delta function, and $\left(r_{s}^{*},r_{i}^{*}\right)$ denotes the prescribed target reliability point.

**Random PriorAug.** The reliability pair is randomly sampled from the reliability space:

(18) $$q\left(r_{s},r_{i}\right)=U\left(0,1\right)\times U\left(0,1\right),$$

or equivalently,

(19) $$r_{s},r_{i}∼U\left(0,1\right).$$

Here, U(0,1) denotes the uniform distribution on [0,1]. Random sampling provides broader coverage over the reliability space and reduces over-specialization to a single operating point.

The training objective follows the underlying traffic generation model:

(20) $$L=L_{\mathrm{gen}}+\lambda L_{\mathrm{reg}},$$

where $L_{\mathrm{gen}}$ is the original generation loss and $L_{\mathrm{reg}}$ denotes the regularization term inherited from the backbone architecture, while λ is the weighting coefficient of the regularization term.

Since PriorAug modifies perceived prior conditions during offline training and does not introduce an additional learnable inference module or iterative decoding stage, the trained generator follows the standard inference procedure of the underlying backbone.

## 4. Experiments

This section evaluates the proposed PriorAug framework on the Waymo Open Motion Dataset. We assess generation quality from three perspectives: distribution fidelity, robustness across shifted-prior and traffic density conditions, and physical plausibility. We further analyze the reliability landscape over the proposed two-dimensional reliability space.

### 4.1. Experimental Settings

**Dataset and task.** The proposed framework is instantiated on the Waymo Open Motion Dataset (WOMD), a large-scale autonomous driving motion dataset collected using heterogeneous sensing systems, including cameras, LiDARs, localization modules, and high-definition mapping pipelines [21]. The dataset is publicly released through the official Waymo Open Dataset platform [22]. Rather than treating WOMD as a generic trajectory dataset, we interpret it as a structured scene representation reconstructed from multi-sensor perception, which naturally matches the sensor-perceived prior formulation introduced in Section 3.

Following the traffic scenario initialization setting, the model receives partial scene context and generates the initial states of the remaining traffic participants, including position, heading angle, speed, and physical size.

**Reliability evaluation protocol.** To validate robustness under continuously varying perception conditions, all evaluations are conducted within the proposed two-dimensional reliability space $\left(r_{s},r_{i}\right)$, where $r_{s}$ and $r_{i}$ denote structural reliability and interaction reliability, respectively.

Structural reliability is controlled through the proposed structural prior operator by masking perceived structural information, while interaction reliability is adjusted through the interaction prior operator using reliability-aware interaction interpolation. Besides evaluating representative operating points, we further investigate coverage-oriented training over the entire reliability space to examine robustness under continuously changing sensor-perceived priors.

**Compared methods.** We compare the proposed framework with deterministic-prior baselines and additional training-strategy baselines. TrafficGen serves as the structural prior baseline operating at $\left(r_{s},r_{i}\right)=\left(1,N/A\right)$, where the model uses structural information but does not contain an explicit interaction reliability branch. Interaction-enhanced TrafficGen serves as the full-prior baseline operating at (1,1), where both structural and interaction priors are fully preserved.

To further isolate the effect of reliability-aware training, we introduce three additional controlled baselines. TrafficGen-MapRand randomizes structural reliability during training on the original TrafficGen backbone. Since TrafficGen has no explicit interaction branch, $r_{i}$ is not applicable to this baseline. IE-TrafficGen-MapRand randomizes only structural reliability while keeping interaction reliability fixed at $r_{i}=1$. IE-TrafficGen-InterRand randomizes only interaction reliability while keeping structural reliability fixed at $r_{s}=1$. These baselines are designed to distinguish joint reliability space training from structural-only randomization and interaction-only randomization.

For the proposed framework, PriorAug_01 represents the weak-structure fixed operating point (0,1), PriorAug_0505 corresponds to the target shifted operating point (0.5,0.5), and PriorAug_Rand denotes the coverage-oriented variant trained by jointly sampling structural and interaction reliability conditions from the proposed two-dimensional reliability space.

Recent traffic generation methods such as UniGen and SceneControl are also discussed in Section 2.1. However, their original formulations target joint initial-state-and-trajectory generation or controllable diffusion-based scene generation and use different output targets, conditioning interfaces, decoding procedures, and evaluation protocols. Their originally reported results are therefore not directly comparable with the initialization-only reliability-shift protocol adopted in this work. A direct numerical comparison would require architecture-specific reimplementation and retraining rather than applying the original methods unchanged.

To isolate the contribution of reliability-aware training under a unified architecture and evaluation protocol, we therefore introduce TrafficGen-MapRand, IE-TrafficGen-MapRand, and IE-TrafficGen-InterRand as controlled baselines. These variants share the same backbone, data split, decoding procedure, and inference budget, and differ only in the dimensions of reliability randomized during training.

**Evaluation metrics.** To comprehensively evaluate traffic scenario initialization, we consider three complementary aspects: distribution fidelity, robustness across scene complexity, and physical plausibility.

**MMD-based distribution fidelity.** Distribution fidelity is evaluated using Maximum Mean Discrepancy (MMD) between the generated and recorded traffic-attribute distributions. The metric is computed independently for each scene and each attribute a∈{speed,position,heading,size}, and is subsequently aggregated over all valid evaluation scenes.

The recorded samples are drawn from the held-out WOMD evaluation split and serve only as the empirical reference for offline benchmarking. No additional real-world data are collected for the generated scenes, and these reference samples are not required by the generator during deployment.

For scene *s* and attribute *a*, let

(21) $$X_{s}^{a}={\left\{x_{s,i}^{a}\right\}}_{i=1}^{n_{s}},\;Y_{s}^{a}={\left\{y_{s,j}^{a}\right\}}_{j=1}^{n_{s}},\;Z_{s}^{a}=X_{s}^{a}\cup Y_{s}^{a},$$

where $X_{s}^{a}$ and $Y_{s}^{a}$ are the recorded and generated samples, respectively, and $|Z_{s}^{a}|=N_{s}=2n_{s}$.

The adaptive kernel scale is computed from the average off-diagonal squared Euclidean distance between the pooled samples:

(22) $$b_{s,a}=\frac{1}{N_{s}\left(N_{s}-1\right)}\sum_{p\neq q}{∥z_{s,p}^{a}-z_{s,q}^{a}∥}_{2}^{2}.$$

A single Gaussian kernel is then defined as

(23) $$k_{s,a}\left(u,v\right)=\exp\left(-\frac{{∥u-v∥}_{2}^{2}}{b_{s,a}}\right).$$

Under the conventional parameterization $k\left(u,v\right)=\exp[-∥u-v{∥}_{2}^{2}/\left(2\sigma_{s,a}^{2}\right)]$, this is equivalent to $\sigma_{s,a}=\sqrt{b_{s,a}/2}$.

The scene-level biased empirical squared MMD is calculated as

(24) $$\begin{array}{cc} {\hat{\mathrm{MMD}}}_{s,a}^{2}= & \frac{1}{n_{s}^{2}}\sum_{i=1}^{n_{s}}\sum_{i^{\prime }=1}^{n_{s}}k_{s,a}\left(x_{s,i}^{a},x_{s,i^{\prime }}^{a}\right)\\ \; & +\frac{1}{n_{s}^{2}}\sum_{j=1}^{n_{s}}\sum_{j^{\prime }=1}^{n_{s}}k_{s,a}\left(y_{s,j}^{a},y_{s,j^{\prime }}^{a}\right)\\ \; & -\frac{2}{n_{s}^{2}}\sum_{i=1}^{n_{s}}\sum_{j=1}^{n_{s}}k_{s,a}\left(x_{s,i}^{a},y_{s,j}^{a}\right). \end{array}$$

Let $S_{a}$ denote the set of valid evaluation scenes for attribute *a*. The squared discrepancy is first averaged over the evaluation scenes, after which the square root is taken:

(25) $${\mathrm{MMD}}_{a}=\sqrt{\max\left(\frac{1}{|S_{a}|}\sum_{s\in S_{a}}{\hat{\mathrm{MMD}}}_{s,a}^{2},0\right)}.$$

The same adaptive bandwidth estimation rule and kernel configuration are applied independently to every scene, attribute, and compared method. Lower MMD values indicate closer agreement between generated and recorded traffic-attribute distributions.

For the reliability-landscape analysis, the average MMD over the four attributes is further reported as

(26) $$\mathrm{AvgMMD}=\frac{1}{4}\sum_{a\in \{\mathrm{speed},\mathrm{position},\mathrm{heading},\mathrm{size}\}}{\mathrm{MMD}}_{a}.$$

**Scene-complexity robustness.** The fixed subset is divided into four buckets according to the number of traffic participants: 8–10, 11–15, 16–20, and 21+.

**Overlap-based physical plausibility.** Physical plausibility is evaluated using SceneOverlap and PairOverlap. For a generated scene *s*, let $B_{i}^{s}$ denote the oriented bounding box of agent *i*, and define the pairwise overlap indicator as

(27) $$o_{ij}^{s}=I\left(\mathrm{Area}(B_{i}^{s}\cap B_{j}^{s})>\epsilon \right),\;i<j,$$

where I(·) denotes the indicator function, and $\epsilon =10^{-6}\;m^{2}$ is used to avoid numerical artifacts. Let S denote the set of evaluation scenes. SceneOverlap measures the percentage of scenes containing at least one overlapping vehicle pair:

(28) $$\mathrm{SceneOverlap}=\frac{100}{\left|S\right|}\sum_{s\in S}I\left(\sum_{i<j}o_{ij}^{s}>0\right).$$

Let $A_{s}$ denote the number of agents in scene *s*. PairOverlap measures the average percentage of overlapping vehicle pairs within each scene:

(29) PairOverlap=100|S|∑s∈S1As2∑i<joijs.

Lower SceneOverlap and PairOverlap values indicate more physically feasible traffic initialization results.

Unless otherwise specified, full-set MMD is computed on all 10,000 evaluation scenarios, containing more than 60,000 valid samples after preprocessing. For computationally intensive shifted-prior, bucket-wise, and overlap analyses, 1000 scenarios were selected by scenario-level simple random sampling with a fixed seed and then processed using the same sample-construction pipeline, yielding more than 6000 valid samples. No stratification by sample count or density bucket was used. The same fixed subset was applied to every method, and subset results are used only to compare relative method behavior under identical conditions, while the main distribution fidelity results remain based on the complete set.

**Implementation details.** All reproduced baselines are trained using the same training–validation split and evaluated under an identical inference protocol. Repeat sampling, collision retry, and all decoding hyperparameters are kept unchanged across different methods so that performance differences primarily reflect modeling capability rather than inference budget.

The models are trained for 100 epochs using a batch size of 32 with the Adam optimizer. The initial learning rate is $3\times 10^{-4}$, the weight decay is $4\times 10^{-4}$, and ten data-loading workers are employed throughout training. All experiments are conducted on a single NVIDIA GeForce RTX 4090 GPU (NVIDIA Corporation, Santa Clara, CA, USA) using PyTorch 1.10.0 with CUDA 11.3 and PyTorch Lightning 1.5.10.For the principal PriorAug models, three random seeds are adopted and the average performance is reported whenever applicable.

PriorAug is designed as an offline training-time augmentation strategy rather than an end-to-end real-time autonomous driving framework. During training, its reliability operators perform element-wise structural masking and interaction-context interpolation. These operations introduce no additional learnable inference modules or iterative decoding stages. After training, the generator follows the standard inference procedure of the underlying backbone. An end-to-end real-time evaluation covering upstream sensing, tracking, mapping, and closed-loop simulation is outside the scope of this study.

We first evaluate whether PriorAug improves traffic scenario initialization under standard and shifted-prior conditions. The analysis covers full-set distribution fidelity, density-stratified robustness, and overlap-based physical plausibility.

### 4.2. Distribution Fidelity

Table 1 reports the overall distribution fidelity evaluated by Maximum Mean Discrepancy (MMD) on four fundamental traffic attributes, including speed, position, heading, and size. Lower values indicate better agreement with the real traffic distribution.

**Table 1:** Full-set MMD comparison under matched training–test reliability settings. All results are computed on the complete evaluation set. Lower values are better.

Model	Training	Test	Speed	Position	Heading	Size
TrafficGen	(1,N/A)	(1,N/A)	0.1602	0.1192	0.1189	0.0932
Interaction-enhanced TrafficGen	(1,1)	(1,1)	0.0634	0.0740	0.0697	0.0665
PriorAug_01	(0,1)	(0,1)	0.1143±0.0002	0.1144±0.0004	0.1141±0.0007	0.0734±0.0002
PriorAug_0505	(0.5,0.5)	(0.5,0.5)	0.0732	0.0766	0.0747	0.0791
PriorAug_Rand	(Rand,Rand)	(Rand,Rand)	0.0734	0.0753	0.0741	0.0747

Note: For fixed-point models, the test condition matches the corresponding training reliability point. “Rand” denotes reliability randomization during training and evaluation. For PriorAug_Rand, $r_{s}$ and $r_{i}$ are independently sampled from U(0,1). For the original TrafficGen backbone, $r_{i}$ is marked as N/A because this backbone does not include an explicit interaction reliability branch. Results under the common shifted condition $\left(r_{s},r_{i}\right)=\left(0.5,0.5\right)$ are reported in Table 2.

**Table 2:** Subset MMD comparison under the common shifted operating point $\left(r_{s},r_{i}\right)=\left(0.5,0.5\right)$. All methods are evaluated on the same fixed scenario-level subset. Lower values are better.

Model	Training	Test	Speed	Position	Heading	Size
TrafficGen	(1,N/A)	(0.5,N/A)	0.3615	0.3786	0.1933	0.7750
TrafficGen-MapRand	(Rand,N/A)	(0.5,N/A)	0.2756	0.2369	0.1736	0.1889
Interaction-enhanced TrafficGen	(1,1)	(0.5,0.5)	0.7264	0.3263	0.3586	0.1679
IE-TrafficGen-MapRand	(Rand,1)	(0.5,0.5)	0.4936	0.2376	0.2876	0.1267
IE-TrafficGen-InterRand	(1,Rand)	(0.5,0.5)	0.0898	0.0962	0.0879	0.0802
PriorAug_01	(0,1)	(0.5,0.5)	0.1102	0.1117	0.1112	0.0739
PriorAug_0505	(0.5,0.5)	(0.5,0.5)	0.0706	0.0744	0.0745	0.0791
PriorAug_Rand	(Rand,Rand)	(0.5,0.5)	0.0706	0.0753	0.0696	0.0771

Note: “Rand” denotes reliability randomization during training. For the original TrafficGen backbone, $r_{i}$ is marked as N/A because this backbone does not include an explicit interaction reliability branch. All methods are evaluated on the same fixed scenario-level subset under the target shifted condition.

Table 1 shows that Interaction-enhanced TrafficGen achieves the strongest performance at its native full-prior operating point, while PriorAug_Rand retains competitive full-set fidelity despite being trained over a broader reliability space. Differences in Size MMD are smaller than those in Speed, Position, and Heading, suggesting that object dimensions are less sensitive to prior-reliability variation. These matched-condition results establish that robustness-oriented training does not substantially sacrifice native distribution fidelity; shifted-condition robustness is examined next.

### 4.3. Robustness Under Prior Shift and Scene Complexity

Overall distribution fidelity may conceal different behaviors under traffic scenes with varying complexity. In practical autonomous driving systems, dense scenes usually introduce stronger occlusions, denser interactions, more difficult object association, and higher behavior estimation uncertainty. Therefore, we further evaluate robustness across different traffic density levels.

Before bucket-wise analysis, Table 2 reports the overall MMD results on the fixed one-tenth evaluation subset under the common shifted operating point $\left(r_{s},r_{i}\right)=\left(0.5,0.5\right)$. This table includes additional controlled baselines that isolate the effects of structural-only and interaction-only reliability randomization.

The results first show that deterministic-prior baselines degrade clearly once the perceived-prior condition shifts away from their native operating points. TrafficGen, which is trained with full structural information and no explicit interaction branch, suffers from large errors under partial structural reliability. TrafficGen-MapRand reduces the MMD values across all four attributes, indicating that structural-reliability randomization improves robustness on the structure-only backbone. However, its performance remains clearly worse than the interaction-aware PriorAug variants, suggesting that structural randomization alone is insufficient for robust initialization under the representative shifted condition.

For the interaction-enhanced backbone, IE-TrafficGen-MapRand improves over Interaction-enhanced TrafficGen, showing that randomizing structural reliability can partially alleviate structural prior shift. Nevertheless, it still produces relatively large errors because the interaction branch remains trained under full interaction reliability and is therefore mismatched when $r_{i}$ decreases at test time. In contrast, IE-TrafficGen-InterRand substantially reduces the MMD values by exposing the model to varying interaction reliability during training. This indicates that interaction-pathway calibration is an important source of robustness at the target shifted point.

Most importantly, PriorAug_Rand achieves the lowest average MMD among the compared training strategies. Compared with IE-TrafficGen-InterRand, joint structural–interaction randomization further reduces the MMD values across all four attributes. This confirms that single-dimension randomization cannot fully replace joint coverage over the two-dimensional reliability space. The proposed PriorAug training strategy therefore improves robustness not merely by adding generic random perturbations, but by explicitly covering the coupled variation of structural and interaction reliability.

**Figure 3:**
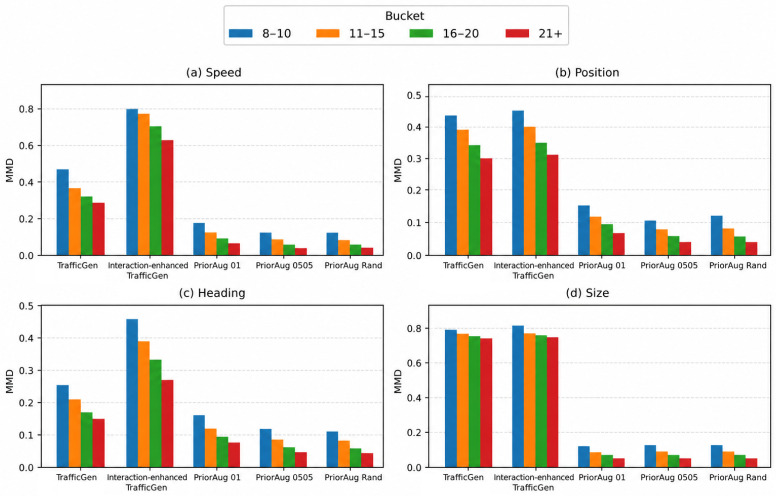
Bucket-wise MMD comparison under different traffic densities at the shifted operating point $\left(r_{s},r_{i}\right)=\left(0.5,0.5\right)$.

To further examine how this degradation varies with traffic complexity, we divide the fixed evaluation subset into four density buckets, namely 8–10, 11–15, 16–20, and 21+ traffic participants. The bucket-wise results are shown in Figure 3.

Across all density buckets, the PriorAug variants retain substantially lower MMD than the deterministic-prior baselines, particularly for Speed, Position, and Heading, demonstrating that reliability-aware training remains effective as scene complexity increases.

### 4.4. Physical Plausibility Analysis

While distribution fidelity measures statistical consistency with real traffic, physically feasible traffic scenario initialization requires that generated traffic participants also satisfy fundamental geometric constraints. In practical autonomous driving applications, unrealistic overlaps or collisions among initialized vehicles may lead to physically invalid simulation states, thereby reducing the reliability of downstream planning and simulation. Therefore, besides distribution matching, physical plausibility serves as another important criterion for evaluating traffic scenario initialization.

Table 3 reports the overlap evaluation using SceneOverlap and PairOverlap. Lower values indicate fewer geometric conflicts and therefore better physical feasibility.

Table 3 shows that all PriorAug variants substantially reduce geometric conflicts relative to the deterministic-prior baselines. PriorAug_01 produces the lowest overlap rates, PriorAug_0505 is closest to the ground-truth reference, and PriorAug_Rand also remains near the ground-truth level.

Because no collision-related objective is used during training, these results suggest that exposure to varying structural and interaction reliability improves the learned spatial relationships among traffic participants. The overlap metrics therefore complement MMD by confirming that the robustness improvement also extends to physical plausibility.

### 4.5. Reliability Landscape Analysis

The shifted-point and bucket-wise results provide evidence at representative reliability conditions.

**Figure 4:**
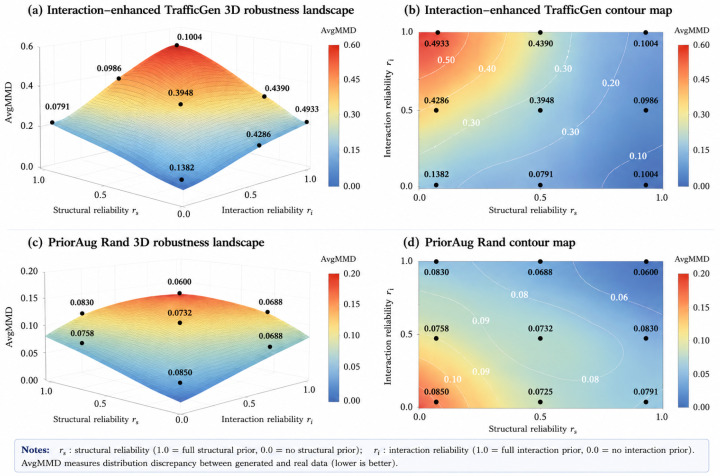
AvgMMD landscapes over structural and interaction reliability. AvgMMD denotes the average MMD across speed, position, heading, and size. The reliability axes are structural reliability $r_{s}$ and interaction reliability $r_{i}$. Panels (**a**,**b**) show the three-dimensional surface and contour map, respectively, for Interaction-enhanced TrafficGen, whereas panels (**c**,**d**) show the corresponding results for PriorAug_Rand. Lower values indicate better point-wise fidelity, while flatter surfaces and smoother contours indicate stronger robustness to reliability shifts.

To further examine global robustness, we evaluate the trained models over the two-dimensional reliability space and visualize the resulting AvgMMD landscapes in Figure 4.

Interaction-enhanced TrafficGen achieves its best performance near its native full-prior configuration $\left(r_{s},r_{i}\right)=\left(1,1\right)$, but its performance rapidly deteriorates when either structural or interaction reliability decreases. This forms a steep and localized low-error region, indicating strong dependence on one deterministic-prior configuration. In contrast, PriorAug_Rand produces a much flatter landscape, with low-error regions extending over a broader range of reliability combinations. This demonstrates that coverage-oriented prior augmentation encourages smoother conditional distribution learning under varying perception conditions.

To quantify local robustness, we compute the Local Reliability Sensitivity (LRS) around representative operating points. Let $f(r_{s},r_{i})$ denote the average MMD over the evaluated attributes. The local sensitivities along the structural and interaction dimensions are defined as

(30) $${\mathrm{LRS}}_{s}=\left|\frac{\partial f(r_{s},r_{i})}{\partial r_{s}}\right|,\;{\mathrm{LRS}}_{i}=\left|\frac{\partial f(r_{s},r_{i})}{\partial r_{i}}\right|.$$

Because the reliability grid interval is ∆=0.5, central differences are used for interior points and forward differences for boundary points.

For Interaction-enhanced TrafficGen, evaluated around its native operating point (1,1), the estimated sensitivities are ${\mathrm{LRS}}_{s}=0.0390$ and ${\mathrm{LRS}}_{i}=0.0338$. For PriorAug_Rand, evaluated around (0.5,0.5), the sensitivities decrease to ${\mathrm{LRS}}_{s}=0.0070$ and ${\mathrm{LRS}}_{i}=0.0075$. Thus, PriorAug_Rand reduces local sensitivity by approximately 5.6× in the structural direction and 4.5× in the interaction direction.

The flatter surface and substantially lower LRS values of PriorAug_Rand show that its improvement extends beyond isolated test points and reflects a smoother response throughout the reliability space.

### 4.6. Sensitivity of the Interaction Reliability Operator

To test whether the generator responds to the effective interaction contribution defined by the interaction reliability operator, we compare $r_{i}=1.0$ and $r_{i}=0.5$ at $r_{s}=0.5$. As shown in Table 4, reducing $r_{i}$ consistently lowers Speed MMD across all density buckets, while Position MMD remains similar. Thus, $r_{i}$ affects generation behavior rather than acting as a purely notational interpolation coefficient. This experiment is a sensitivity analysis of interaction strength, not a full ablation of alternative neutral endpoints, which would require retraining separate zero-vector, dataset-mean, learnable-embedding, and base-context models.

## 5. Discussion

The results show that deterministic-prior generators can perform well near their native operating conditions but become sensitive when structural or interaction reliability shifts. In contrast, PriorAug maintains competitive matched-condition fidelity and improves shifted, density-stratified, landscape, and overlap-based performance. The controlled baselines further indicate that joint structural–interaction randomization provides broader robustness than randomizing either dimension alone.

This work has two main limitations. First, reliability is controlled by predefined degradation operators rather than estimated directly from a real perception system. Second, the shifted-prior, bucket-wise, and overlap analyses use a fixed one-tenth subset because of computational cost. Future work will connect $r_{s}$ and $r_{i}$ to perception confidence, localization uncertainty, online-map quality, and tracking confidence, and extend the evaluation to larger-scale, long-horizon, and closed-loop simulation.

## 6. Conclusions

This paper introduced PriorAug, which models traffic scenario initialization over a two-dimensional space of structural and interaction reliability. Reliability-conditioned training preserves competitive full-set distribution fidelity while improving robustness under shifted-prior conditions and varying traffic densities, as well as the physical plausibility of generated initial states. The smoother reliability landscape and lower local sensitivity of PriorAug_Rand further show that coverage-oriented training reduces over-specialization to isolated deterministic operating points. These results support reliability-aware prior augmentation as a practical approach to robust traffic scenario initialization under uncertain sensor-perceived conditions.

## Figures and Tables

**Table 3:** Physical plausibility evaluation under the common shifted test condition $\left(r_{s},r_{i}\right)=\left(0.5,0.5\right)$ on the same fixed scenario-level subset used in Table 2. Lower values indicate fewer geometric conflicts and better physical plausibility.

Model	Training Setting	Test Setting	SceneOverlap (%)		PairOverlap (%)	
			Pred.	GT	Pred.	GT
TrafficGen	(1,N/A)	(0.5,N/A)	9.616	1.821	0.095025	0.014166
Interaction-enhanced TrafficGen	(1,1)	(0.5,0.5)	30.053	1.821	0.348252	0.014166
PriorAug_01	(0,1)	(0.5,0.5)	1.008	1.821	0.008375	0.014166
PriorAug_0505	(0.5,0.5)	(0.5,0.5)	1.761	1.821	0.014993	0.014166
PriorAug_Rand	(Rand,Rand)	(0.5,0.5)	1.550	1.821	0.012511	0.014166

Note: All methods use the same fixed subset and the test condition of Table 2. For TrafficGen, $r_{i}$ is N/A. Generation uses repeat=10 and max_retry=5; the ground-truth values are computed on the same subset.

**Table 4:** Sensitivity to interaction strength under the representative shifted structural prior condition $r_{s}=0.5$. Lower values indicate better distribution fidelity.

Bucket	Speed MMD		Position MMD	
	$r_{i}=1.0$	$r_{i}=0.5$	$r_{i}=1.0$	$r_{i}=0.5$
8–10	0.1677±0.0225	0.1483±0.0175	0.1567±0.0081	0.1606±0.0092
11–15	0.1320±0.0321	0.1070±0.0146	0.1178±0.0058	0.1203±0.0108
16–20	0.1087±0.0299	0.0797±0.0110	0.0910±0.0031	0.0935±0.0071
21+	0.0818±0.0305	0.0536±0.0083	0.0706±0.0049	0.0701±0.0045

## Data Availability

The Waymo Open Motion Dataset analyzed in this study is publicly available from the Waymo Open Dataset platform subject to its data license and access requirements. No new public dataset was created in this study.

## Abbreviations

The following abbreviations are used in this manuscript:

AV	Autonomous vehicle
HD map	High-definition map
ITS	Intelligent transportation system
MMD	Maximum mean discrepancy
WOMD	Waymo Open Motion Dataset

## References

[1] Tan S., Wong K., Wang S., Manivasagam S., Ren M., Urtasun R. (2021). SceneGen: Learning to Generate Realistic Traffic Scenes. Proceedings of the IEEE/CVF Conference on Computer Vision and Pattern Recognition (CVPR), Nashville, TN, USA, 20–25 June 2021.

[2] Feng L., Li Q., Peng Z., Tan S., Zhou B. TrafficGen: Learning to Generate Diverse and Realistic Traffic Scenarios. Proceedings of the IEEE International Conference on Robotics and Automation (ICRA).

[3] Mahjourian R., Mu R., Likhosherstov V., Mougin P., Huang X., Messias J., Whiteson S. UniGen: Unified Modeling of Initial Agent States and Trajectories for Generating Autonomous Driving Scenarios. Proceedings of the IEEE International Conference on Robotics and Automation (ICRA).

[4] Lu J., Wong K., Zhang C., Suo S., Urtasun R. (2024). SceneControl: Diffusion for Controllable Traffic Scene Generation. Proceedings of the IEEE International Conference on Robotics and Automation (ICRA), Yokohama, Japan, 13–17 May 2024.

[5] Tan S., Ivanovic B., Weng X., Pavone M., Krahenbuhl P. (2023). Language Conditioned Traffic Generation. Proceedings of the Conference on Robot Learning (CoRL).

[6] Elghazaly G., Frank R., Harvey S., Safko S. (2023). High-Definition Maps: Comprehensive Survey, Challenges and Future Perspectives. IEEE Open J. Intell. Transp. Syst..

[7] Asrat K.T., Cho H.-J. (2024). A Comprehensive Survey on High-Definition Map Generation and Maintenance. ISPRS Int. J. Geo-Inf..

[8] Zang A., Xu R., Zhou F. (2024). Data Issues in High-Definition Maps: A Survey. ACM Trans. Spat. Algorithms Syst..

[9] Hui N., Jiang Z., Cai Z., Ying S. (2024). Vision-HD: Road Change Detection and Registration Using Images and High-Definition Maps. Int. J. Geogr. Inf. Sci..

[10] Zhang G., Lin J., Wu S., Song Y., Luo Z., Xue Y., Lu S., Wang Z. (2023). Online Map Vectorization for Autonomous Driving: A Rasterization Perspective. Advances in Neural Information Processing Systems.

[11] Gao J., Ma C., Li M., Dai S., Zhu Y., Wang Y., Yang B. VectorNet: Encoding HD Maps and Agent Dynamics from Vectorized Representation. Proceedings of the IEEE/CVF Conference on Computer Vision and Pattern Recognition (CVPR).

[12] Liang M., Yang B., Hu R., Wang Y., Urtasun R. (2020). Learning Lane Graph Representations for Motion Forecasting. Computer Vision–ECCV 2020.

[13] Gupta A., Johnson J., Fei-Fei L., Savarese S., Alahi A. Social GAN: Socially Acceptable Trajectories with Generative Adversarial Networks. Proceedings of the IEEE/CVF Conference on Computer Vision and Pattern Recognition (CVPR).

[14] Salzmann T., Ivanovic B., Chakravarty P., Pavone M. (2020). Trajectron++: Dynamically-Feasible Trajectory Forecasting with Heterogeneous Data. Computer Vision–ECCV 2020.

[15] Yuan Y., Weng X., Ou Y., Kitani K. AgentFormer: Agent-Aware Transformers for Socio-Temporal Multi-Agent Forecasting. Proceedings of the IEEE/CVF International Conference on Computer Vision (ICCV).

[16] Tobin J., Fong R., Ray A., Schneider J., Zaremba W., Abbeel P. (2017). Domain Randomization for Transferring Deep Neural Networks from Simulation to the Real World. Proceedings of the IEEE/RSJ International Conference on Intelligent Robots and Systems (IROS), Vancouver, BC, Canada, 24–28 September 2017.

[17] Sadeghi F., Levine S. CAD2RL: Real Single-Image Flight without a Single Real Image. In Proceedings of Robotics: Science and Systems (RSS).

[18] Peng X.B., Andrychowicz M., Zaremba W., Abbeel P. (2018). Sim-to-Real Transfer of Robotic Control with Dynamics Randomization. Proceedings of the IEEE International Conference on Robotics and Automation (ICRA), Brisbane, Australia, 21–25 May 2018.

[19] Rajeswaran A., Ghotra S., Ravindran B., Levine S. EPOpt: Learning Robust Neural Network Policies Using Model Ensembles. Proceedings of the International Conference on Learning Representations (ICLR).

[20] Pinto L., Davidson J., Sukthankar R., Gupta A. (2017). Robust Adversarial Reinforcement Learning. Proceedings of the International Conference on Machine Learning (ICML), Sydney, Australia, 6–11 August 2017.

[21] Ettinger S., Cheng S., Caine B., Liu C., Zhao H., Pradhan S., Chai Y., Sapp B., Qi C.R., Zhou Y. (2021). Large Scale Interactive Motion Forecasting for Autonomous Driving: The Waymo Open Motion Dataset. Proceedings of the IEEE/CVF International Conference on Computer Vision (ICCV), Montreal, QC, Canada, 10–17 October 2021.

[22] Waymo Open Dataset. https://waymo.com/open/.

